# The Effect of Adrenaline on the Mineral and Trace Element Status in Rats

**DOI:** 10.1515/biol-2019-0018

**Published:** 2019-05-17

**Authors:** Svitlana Shkurashivska, Hanna Ersteniuk

**Affiliations:** 1 Ivano-Frankivsk College of Physical Education, 142a Mazepa St., 76026, Ivano-Frankivsk, Ukraine; 2 Ivano-Frankivsk National Medical University, 2 Halytska St., 76018, Ivano-Frankivsk, Ukraine

**Keywords:** adrenaline, copper (Cu), zinc (Zn), magnesium (Mg), calcium (Ca), rat

## Abstract

Up until now, changes in biochemical and physiological parameters occurring a long time after stress are not yet elucidated. This is particularly the case for metals, some of which may considerably influence other branches of metabolism, such as bioenergetics and antioxidant defense. The aim of the current study was to investigate changes in levels of minerals (calcium and magnesium) and trace elements (copper and zinc) in erythrocytes and the liver of rats injected once or twice (modeling repeated stress) with adrenaline. The tissues were sampled 0.5 and 24 hours after the injection. A single injection of adrenaline in rats led to a dramatic increase in the levels of zinc (Zn), magnesium (Mg), and calcium (Ca) in their erythrocytes and liver, without a return to the control level (unstressed animals) after 24 hours. The levels of copper (Cu) increased 0.5 hour after a single adrenaline injection in erythrocytes and the liver, but returned to the control level after 24 hours. Double injection of rats with adrenaline led to an increase in the levels of Cu and Zn in their erythrocytes, and Mg in the liver, without a return to the control level after 24 hours. On the other hand, the double injection led to a drastic but transient increase in levels of Mg and Ca in erythrocytes, and Cu, Zn, and Ca in the liver. Thus, injection with adrenaline results in dramatic changes in levels of minerals and trace elements, which do not return to the control level after stress. Low doses of adrenaline lead to more stable changes in levels of essential metals.

## 1 Introduction

The response of living organisms to stresshas been studied intensively since the middle of the 20^th^ century. In multicellular organisms, this response is coordinated by a number of hormones, in particular, adrenaline, noradrenaline and cortisol [1, 2]. Activation of the adrenaline signaling pathway is one of the most important elements of the body’s response to stress [1, 2]. The effects of this activation within a short period are currently studied in sufficient detail at the level of cells, tissues and the whole organism. However, the characteristic changes in the physiological and biochemical parameters of the organism for a long time after the release of adrenaline have not been studied sufficiently. The effects of on changes in the concentration of spare and mobile carbohydrates, as well as lipids and their catabolic products – glycerol and fatty acids – have been studied in detail. However, an understanding of the regulation of metabolic processes, in particular the role of minerals and trace elements, is not yet studied in detail.

It is known that adrenaline contributes to the release of calcium ions from the endoplasmic reticulum [3]. In turn, calcium launches a number of other signaling events (e.g., activates calmodulin-dependent kinases) [3]. In addition to calcium, other metals, such as magnesium, copper, and zinc are important, and their transportation to and from cells may vary, depending on the activity of adrenaline signaling [4, 5]. The levels of these metals in cells are determining factors in the regulation of energy metabolism. In particular, magnesium is a cofactor in many reactions in which adenosine triphosphate or its derivatives are involved [6]. Copper is a part of cytochrome oxidase, a component of the respiratory chain of mitochondria, and zinc is an activator of lactate dehydrogenase, etc [7]. Both metals are present in copper/zinc superoxide dismutase, an important antioxidant enzyme [7]. Prolonged or repeated stress, influencing metal homeostasis, may subsequently result in changes associated with metal-containing enzymes, including those listed above.

The aim of our research was to find out how adrenaline will affect the content of calcium, magnesium, copper and zinc in the red blood cell mass (RCM) and liver of rats injected once or twice with adrenaline. In our case, the double injection models repeated stress, a situation that frequently occurs naturally. At the same time, we evaluated changes in contents of calcium, magnesium, copper, and zinc at different intervals after the injection of adrenaline.

## 2 Methods

### 2.1 Animals

The experiments were conducted on four-month-old white Wistar rats weighing ca.150-200 g. The animals were kept on a standard vivarium diet, in standard cages, at 20 °C, 50% relative humidity, and 10/14 hours light/dark cycle [8].

**Ethical approval**: The research related to animals use has been complied with all the relevant national regulations and institutional policies for the care and use of animals. The study on rats was approved by permission #53/11 from the Local Ethics Committee of Ivano-Frankivsk National Medical University signed by Professor Iryna Kupnovytska and Docent Svitlana Kaluhina and received on 24.02.2011.

### 2.2 Treatment

The animals of the control and experimental groups were starved during 12 hours before the experiment, receiving only drinking water. For modeling of stress associated with adrenaline release, adrenaline hydrochloride solution was injected once or twice (with a 1 hour interval) at a dose 0.05 mg/kg of body weight [9]. Adrenaline was injected intramuscularly (back leg, inner side). The doses of adrenaline were chosen according to the literature [9, 10].

### 2.3 Sample collection and spectroscopy

The material (blood, liver) was sampled after decapitation under thiopental anesthesia after 30 minutes and 24 hours after the administration of adrenaline. The decapitation was carried out in accordance with the items of Directive 2010/63/EU of the European Union for the protection of animals used for scientific purposes [11]. Heparin was added to the collected blood (7 ml) which was then centrifuged at 1000 × g for 15 min. The pellet of red blood cells was used for subsequent analyses. Pieces of liver (4 g) and red cell mass (RCM) were dried for several hours in ceramic cups at 150 °C, then carbonized at 450 °C in a muffle furnace until white-colored ash was obtained. The ash was then dissolved in 25 ml of ultra-pure nitric acid and slowly dried at 50-450 °C (temperature was increased by 50 °C each 0.5 hour, starting from 50 °C) until homogenous white-colored ash was obtained. The amount of copper, zinc, magnesium, and calcium was determined using an atomic absorption spectrophotometer C/115 PC. Bovine liver was used as a reference for analysis.

### 2.4 Experimental groups

The experimental animals were as follows: Group I – control animals, which were not injected; Group II – animals injected oncewith 0.05 mg/kg adrenaline, followed by a sampling after 30 minutes; Group III – animals injected once with 0.05 mg/kg adrenaline with a further sampling after 24 hours; Group IV – animals injected twice with 0.05 mg/kg adrenaline at intervals of 1 hour with subsequent sampling after 30 minutes; Group V– animals injected twice with 0.05 mg/kg adrenaline with a sampling after 24 hours. There were seven animals (three females and four males) in each group. Quantitative determination of adrenaline was carried out with the help of the colorimetric method [12].

### 2.5 Statistical analysis

Statistical analysis of the data was carried out using the computer program Statistica 7.0. The significance of the difference between the means was estimated by Student’s *t*-test. The differences with *p*-values < 0.05 were considered significant. The values are presented as the mean ± standard error of the mean.

## 3 Results

**Figure 1:**
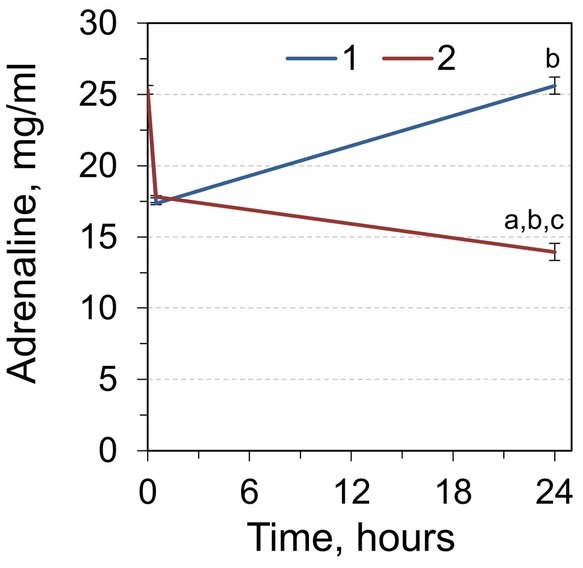
Adrenaline concentration in blood of rats 0, 0.5, and 24 h after injection by single (1) and double dose (2) of adrenaline. ^a^Significantly different from the corresponding value for animals that received single adrenaline injection (*p*< 0.05, Student’s *t*-test, n = 7), ^b^significantly different from the value for the animals sampled 0.5 hours after adrenaline injection, ^c^significantly different from the values of the control group (time point 0).

We found that 30 minutes after a single or double dose of adrenaline injected in Wistar rats, there was a decrease of the level of adrenaline in RCM by 31.5% and 29.6%, respectively, compared to control individuals (Figure 1). The level of adrenaline returned to the control level 24 hours after the single injection. At the same time, the level of adrenaline was 44.9% lower than in the control 24 hours after double injection of the hormone.

**Figure 2:**
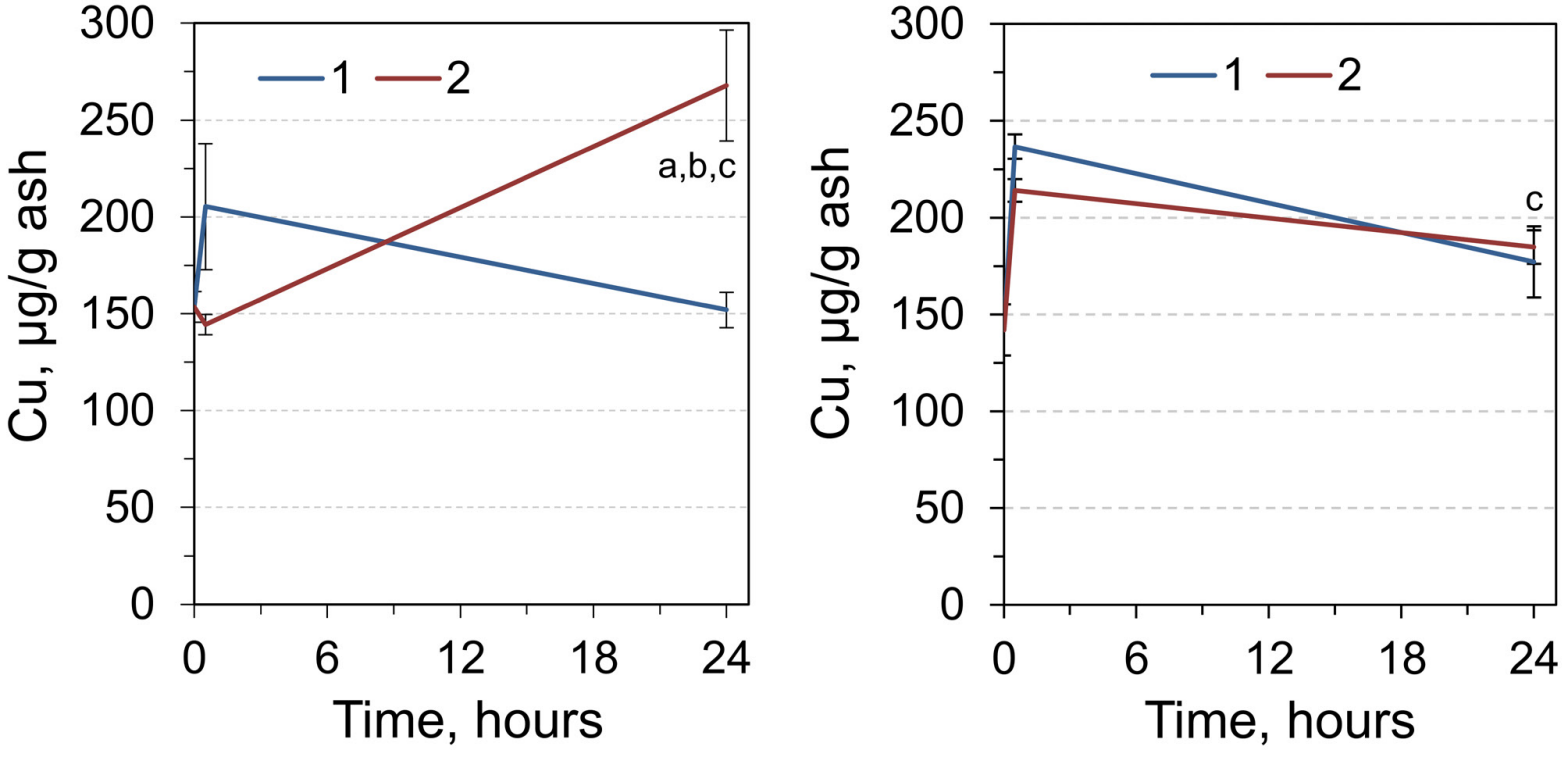
Copper content in red cell mass (RCM) (left) and liver (right) of rats 0, 0.5, and 24 h after injection by single (1) and double dose (2) of adrenaline. ^a^Significantly different from the corresponding value for animals that received single adrenaline injection (*p*< 0.05, Student’s *t*-test, n = 7), ^b^significantly different from the value for the animals sampled 0.5 hours after adrenaline injection, ^c^significantly different from the values of the control group (time point 0).

The level of copper (Cu) in RCM increased by 33.8% 30 minutes after a single dose of adrenaline (Figure 2) and returned to the control level after 24 hours. After the double dose of adrenaline, another tendency was observed. In particular, the concentration of copper in RCM was unchanged 30 minutes after the double injection, but was 1.75-fold higher after 24 hours. Therefore, repeated administration of adrenaline resulted in a significantly higher level of copper (about 1.8-fold) compared to the single administration and a control group.

**Figure 3:**
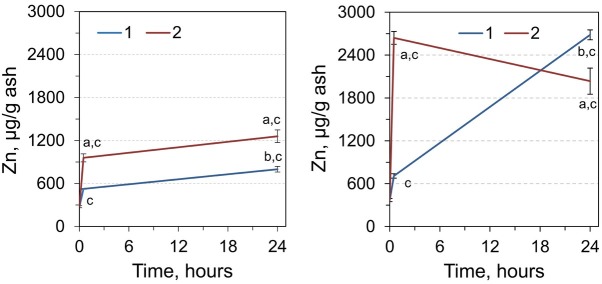
Zinc content in RCM (left) and liver (right) of rats 0, 0.5, and 24 h after injection by single (1) and double dose (2) of adrenaline. ^a^Significantly different from the corresponding value for animals that received single adrenaline injection (*p*< 0.05, Student’s *t*-test, n = 7), ^b^significantly different from the value for the animals sampled 0.5 hours after adrenaline injection, ^c^significantly different from the values of the control group (time point 0).

A clear tendency toward increases in the content of Zn in RCM of all experimental groups should be noted (Figure 3). As such, a 1.91-fold increase was observed 30 minutes after a single dose, and 2.91-fold increase was after 24 hours, compared with the control. We noted an even more significant increase in the level of zinc in RCM after the double dose of adrenaline and sampling after 30 minutes and 24 hours of 3.49 and 4.59 times, respectively.

**Figure 4:**
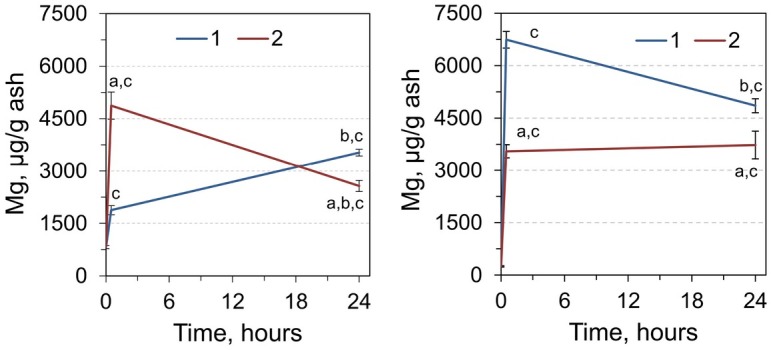
Magnesium content in RCM (left) and liver (right) of rats 0, 0.5, and 24 h after injection by single (1) and double dose (2) of adrenaline. ^a^Significantly different from the corresponding value for animals that received single adrenaline injection (*p*< 0.05, Student’s *t*-test, n = 7), ^b^significantly different from the value for the animals sampled 0.5 hours after adrenaline injection, ^c^significantly different from the values of the control group (time point 0).

An important metal of the anti-stress system is Mg (Figure 4). We found that Mg content in RCM increased by 2.28-fold 30 minutes after a single administration of adrenaline, and by 4.29-fold 24 hours after the administration relative to the control. When the adrenaline was administered twice after 24 hours, the level of Mg in RCM decreased by almost 2-fold compared with the early observation period after 30 minutes but stayed 3.12 times higher compared with the control.

**Figure 5:**
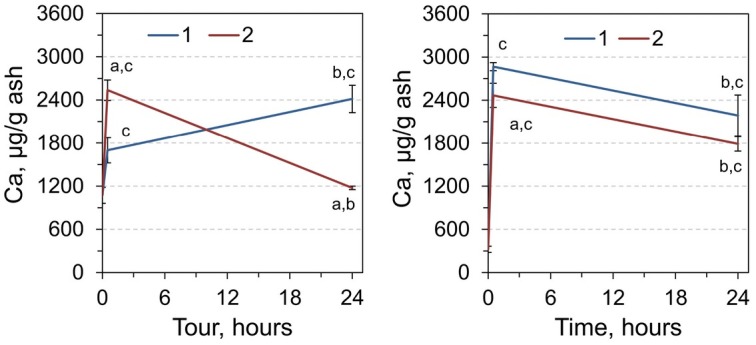
Calcium content in RCM (left) and liver (right) of rats 0, 0.5, and 24 h after injection by single (1) and double dose (2) of adrenaline. ^a^Significantly different from the corresponding value for animals that received single adrenaline injection (*p*< 0.05, Student’s *t*-test, n = 7), ^b^significantly different from the value for the animals sampled 0.5 hours after adrenaline injection, ^c^significantly different from the values of the control group (time point 0).

As for the content of Ca, it should be noted that a true increase was observed in RCM of experimental animals, both in a single and double dose of adrenaline after 30 minutes: a 1.59 times increase and 2.37 times increase, respectively (Figure 5). A slightly different tendency was

observed at the 24 hour point of the experiment: after a single dose of adrenaline, the level of Ca increased by 2.25 times, and at a double-dose, it was almost the same as that of intact animals.

The level of copper in the liver tissues increased significantly after administration of adrenaline in all experimental groups (Figure 2). In a single and double dose of adrenaline after 30 minutes, an increase was observed in 1.67 and 1.51 times, respectively. In addition, one day after single or double administration of adrenaline, copper content was increased by 1.25 and 1.30 times relative to the control.

We have shown the increase of Zn content in all experimental groups relative to the control group. Therefore, Zn content increased by 2-fold 30 minutes after a single dose of adrenaline, and by 7.23-fold one day after the injection. The significant increase in Zn was observed in case of the double injection: after 30 minutes the level of Zn increased by 7.1-fold, and after 24 hours, by 5.48-fold (Figure 3).

Levels of Mg in the liver increased by 27.2 times 30 minutes after a single dose of adrenaline, and by 19.6 times 24 hours after a single dose (Figure 4). A double dose of adrenaline followed by a sampling after 30 minutes and 24 hours was accompanied by an increase of 14.3 and 15.1 times, respectively.

As can be seen from Figure 5, the concentration of calcium in the liver of rats exposed to a single-dose stress and sampling after 30 min increased by 8.87 times, and by 7.63 times after double dose compared with the control group. At the 24 hour point of the experiment, we noted an increase in the content of Ca in the liver of animals of 6.77 times with a single dose of adrenaline, and 5.54 times in the case of a repeated adrenaline injection.

## 4 Discussion

Our studies allowed us to establish that 30 minutes after a single or double dose of adrenaline in experimental animals, there was a decrease in the level of adrenaline in RCM compared with the individuals of the control group, indicating increased inactivation of excess amounts of the hormone, mainly in the liver. It should be noted that with a double dose of the hormone, its level after 24 hours was much lower compared to the control group. It can be assumed that a repeated stress causes a long-term adaptation of the body and restoration of homeostasis is achieved later.

Regarding the reaction – the response of the body to stress, it is worth emphasizing that both single and double doses of adrenaline were accompanied by the redistribution of essential macroelements – Ca and Mg, as well as trace elements Cu and Zn in the organism of the experimental animals.

Regarding the level of copper, it should be noted that increases of this indicator after adrenaline injectionwere observed in RCM and the liver of all experimental groups. From the scientific literature, it is known [13] that under

the influence of adrenaline, Cu starts its mobilization from copper stores. Therefore, an increase of the level of copper with significant pain irritations, infections, intoxications is considered a protective reaction of the organism [14]. The increase in the level of copper in the liver may be due to the transcriptional activation of ceruloplasmin – a copper-containing protein synthesized in the liver whose concentration depends on the hormonal background, which undergoes significant changes in stressful situations and various diseases [13].

The different direction of interconnection between elements in living organisms is well known [15, 16]. Many authors [15, 16] describe the antagonistic effects of Zn in relation to Cu. Our research suggests that under conditions of adrenaline stress in erythrocytes and the liver, the level of zinc increases along with an increase in Cu. A similar pattern was noted in Paneth’s cells and a prostate gland, and in granulocytes of blood at acute stress [17]. Along with this, we have established a slight but significant decrease in Zn in the liver after a double adrenaline administration. Such changes in the content of Zn can be explained as a manifestation of a nonspecific adaptive syndrome of blood cells and the liver, which depends on the frequency and duration of the stress situation. It is known that Zn in athletes decreases after the onset of intensive training and maintained as such for 1 to 2 months after the competition [18].

Observations on the level of Mg have shown that both a single and double dose of adrenaline is accompanied by a decrease of this element in red blood cells. The reduction of the intracellular level of Mg under such circumstances may be due to its release from cells and excretion of urine as described by some authors [19]. On the other hand, for adrenaline stress, we observed a redistribution of this element in the body, which was characterized by an increase of Mg in the liver, which may be due to the high intensity of energy metabolism activated by the action of adrenaline. Since Mg is known as the activator of enzymes that provide energy, it is likely that one of the components of the adaptive complex of reactions to the stress situation is the same, in particular in the early period.

In experimental animals that were put under pressure, hypercalcemia was observed after 30 minutes with a single ordouble dose of adrenaline. After 24 hours, in a single dose, the level of Ca remained high, and in the case of double dose, it was almost the same as in intact animals. Changes of the concentration of Ca in RCM affect its concentration inside the cells due to a malfunction of the calcium pump, calcium-dependent enzymes and regulatory systems. Our data on the calcium content in the liver under adrenaline stress indicated the accumulation of this element, which may be due to the outside entrance through the plasma membranes, as well as through the exit from the intracellular stores. It is known that intracellular Ca is localized predominantly in mitochondria and structures of the endoplasmic reticulum and is released by the action of biologically active compounds, in particular hormones, and mediators. Since Ca is a secondary messenger of the interaction of the sympathoadrenal system of mediators with hepatocyte alpha-adrenergic receptors [3, 20], this may be due to the increase of Ca in the liver.

Thus, our research suggests that in the process of adrenaline stress there is a significant redistribution of life-saving elements, in particular Ca, Mg, Zn, and Cu in red blood cells and liver cells. Considering the involvement of these macro- and microelements in the implementation of hormonal effects (Ca), cell division (Zn), energy metabolism (Mg), antioxidant defense and a range of other metabolic processes, it can be assumed that the adaptation of the organism, in particular in the early period, accompanied by complex changes may predispose the development of dys-microelementosis.
